# An optimized HMGB1 expressed by recombinant rabies virus enhances immunogenicity through activation of dendritic cells in mice

**DOI:** 10.18632/oncotarget.18368

**Published:** 2017-06-05

**Authors:** Zhao Wang, Qian Liang, Yajing Zhang, Jie Yang, Mingming Li, Kunlun Wang, Min Cui, Huanchun Chen, Zhen F. Fu, Ling Zhao, Ming Zhou

**Affiliations:** ^1^ State Key Laboratory of Agricultural Microbiology, Huazhong Agricultural University, Wuhan, China; ^2^ College of Veterinary Medicine, Huazhong Agricultural University, Wuhan, China; ^3^ Key Laboratory of Preventive Veterinary Medicine of Hubei Province, Huazhong Agricultural University, Wuhan, China; ^4^ Department of Pathology, University of Georgia, Athens, GA, USA

**Keywords:** rabies virus, HMGB1, dendritic cells, T follicular helper cells, germinal center B cells

## Abstract

Rabies remains an important public health threat, killing approximately 59,000 people worldwide annually, most of which are from the developing countries of Africa and Asia where dog rabies are endemic. Therefore, developing an affordable and efficacious vaccine for dog-mediated rabies control is needful in these countries. Our previous studies indicated that over-expression of granulocyte-macrophage colony stimulating factor (GM-CSF) or macrophage inflammatory protein-1 (MIP-1α or CCL3) by recombinant rabies virus (rRABV) could enhance the immunogenicity by activating dendritic cells (DCs). In this study, to further characterize the role of activating DCs in RABV immunogenicity, High mobility group box 1 (HMGB1), a highly conserved and non-histone chromosomal protein that can promote DCs maturation and activation, were investigated. The wild-type HMGB1 (HMGB1^wt^) and an optimized HMGB1 (HMGB1^mut^) were individually inserted into the genome of the rRABV strain LBNSE (designated as LBNSE-HMGB1^wt^ and LBNSE-HMGB1^mut^, respectively), and the effect of over-expression of HMGB1 on the immunogenicity of RABV was investigated. The results demonstrated that LBNSE-HMGB1^mut^ could promote significantly more DCs activation, and the recruitment of follicular helper T, germinal center B and plasma cells in vaccinated mice than those immunized with LBNSE-HMGB1^wt^ or parent virus LBNSE. Further investigations suggested that mice vaccinated with LBNSE-HMGB1^mut^ produced significantly higher level of RABV-neutralizing antibodies and offered a better protection than those vaccinated with LBNSE or LBNSE-HMGB1^wt^. Taken together, these data provides a better understanding of the mechanism for HMGB1 as a potential adjuvant in enhancing the immunogenicity of RABV, which would contribute to developing more-efficacious rabies vaccines.

## INTRODUCTION

Rabies, an ancient and fatal zoonotic disease [1, 2], still kills approximately 59,000 persons worldwide each year and brings a potential threat to more than 3 billion people in over 150 countries and territories [3, 4]. The causative agent, rabies virus (RABV), a negative-stranded RNA virus of the genus *Lyssavirus* within the family *Rhabdoviridae*, has a relatively simple and modular genome that encodes five structural proteins in the following order: nucleoprotein (N), phosphorylated protein (P), matrix protein (M), glycoprotein (G), and RNA-dependent RNA polymerase (L) [1, 2]. RABV is a neuro-tropic pathogen, the viral particles from saliva of rabid animal firstly infect the periphery nervous system and subsequently moves along the spinal cord to the brain, causing neuronal dysfunction, which is most likely the major cause of the fatal outcome of rabies [5].

In developed countries, the mass immunization of dogs has already nearly eliminated human rabies. Unfortunately, thousands of persons die from rabies in developing countries each year due to logistical, financial and other unfavorable factors [6]. Although a number of carnivore and bat species act as natural reservoirs of RABV, dogs are the principal vectors in poverty-stricken developing regions, and almost all (99%) human rabies deaths are due to dog bites [7–9]. Therefore, the elimination of canine rabies is the most cost-efficacious mean to control or eliminate rabies in humans [8, 10]. Live-attenuated viruses have recently emerged as a practical and promising way to control rabies [5]. Our previous studies indicated that attenuated RABV expressing cytokines or chemokines could promote immune responses by means of recruiting and/or activating dendritic cells (DCs) [11–15]. Injecting a single dose of these vaccines can induce robust and sustained virus-neutralizing antibody (VNA) production and offer adequate protection to animals against a lethal dose of rabies virus challenge. Hence, enhancing DC activation is an efficacious strategy to promote the humoral immune responses for a rabies vaccine.

After antigens enter the body, DCs capture the antigens and become fully stimulatory, and then they migrate to T-cell areas of secondary lymphoid organs. The interactions of activated DCs with T and B cells is fundamental for the induction of an adaptive immune response [16]. After antigen presentation to T cells, CD4^+^ naïve T cells differentiate into several subtypes: helper T type 1 (Th1), helper T type 2 (Th2), inducible regulatory T (iTReg), interleukin (IL)-17-producing helper T (Th17), or follicular helper T (Tfh) cells [17, 18]. Among these subtypes, Tfh cells are described as non-polarized CD4^+^ T cells expressing the highest levels of chemokine C-X-C motif receptor 5 (CXCR5), programmed cell death protein-1 (PD-1), B-cell lymphoma 6 (BCL-6), interleukin-21 (IL-21), and inducible T-cell co-stimulator (ICOS), but not B lymphocyte-induced maturation protein-1 (Blimp-1) [19, 20]. Sustained contact between Tfh and B cells is necessary for the provision of help to B cells [20]. In addition, Tfh cells are also important for the formation of germinal center (GC). Once GC is formed, Tfh cells are needed to maintain them and regulate the GC B cells differentiation into plasma cells and memory B cells [17, 21, 22].

High mobility group box 1 (HMGB1), a highly conserved and non-histone chromosomal protein, mediates immune responses in the noninfectious inflammatory response [23]. Although HMGB1 is bound to DNA in the nucleus in almost all eukaryotic cells with a extracellular microenvironment baseline [24], it can be rapidly released into the extracellular space when the cell is subjected to stress stimuli [25]. HMGB1 is primarily released from activated monocytes, macrophages [26] and NK cells [27] and behaves as a proinflammatory cytokine. HMGB1 promotes the maturation and activation of DCs [24, 28], induces the migration of DCs into draining lymph nodes (LNs) [29], and is an efficacious endogenous immune adjuvant molecule [30–33].

In this study, to further characterize the role of DCs activation in RABV immunogenicity, an optimized HMGB1 (HMGB1^mut^) was cloned into the attenuated RABV LBNSE strain. The effect of expression of HMGB1^mut^ in the immunogenicity of the RABV was evaluated in a mouse model. The results suggest that over-expression of HMGB1^mut^ promotes RABV-induced humoral immunity by recruiting and/or activating DCs, and then enhancing the recruitment of Tfh, GC B, and plasma cells, suggesting that HMGB1^mut^ could be a good candidate for RABV vaccine.

## RESULTS

### Construction and characterization of rRABVs expressing HMGB1^wt^ or HMGB1^mut^

**Figure 1:**
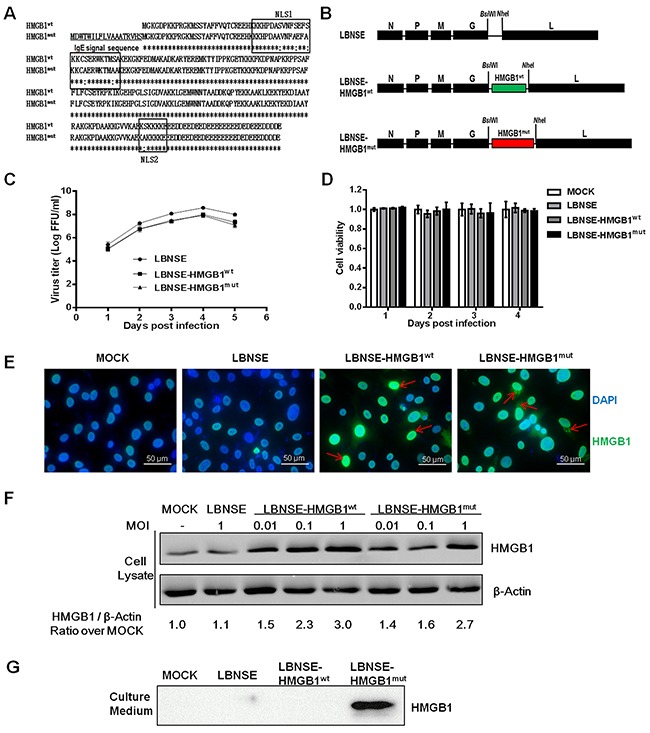
Construction and characterization of the rRABV expressing HMGB1^wt^ or HMGB1^mut^ **(A)** Schematic representation of the strategy for the optimized HMGB1 (HMGB1^mut^). The immunoglobulin G (IgE) signal sequence (underlined) was added before the wild-type HMGB1 (HMGB1^wt^), and then all serine residues in both nuclear localization signals (NLSs) were mutated into alanine residues. **(B)** Schematic diagram for the construction of LBNSE, LBNSE-HMGB1^wt^, and LBNSE-HMGB1^mut^. The mouse HMGB1^wt^ and HMGB1^mut^ were acquired and individually inserted into the genome of LBNSE strain between the G and L gene, and the rRABVs were rescued according to the Materials and Methods. **(C)** A multistep growth curve was depicted. BSR cells were infected with LBNSE, LBNSE-HMGB1^wt^, or LBNSE-HMGB1^mut^ at a multiplicity of infection (MOI) of 0.01, and then incubated at 37°C. The titers were tested as the method described at 1, 2, 3, 4 and 5 dpi. All titrations were undertaken in quadruplicate, and the results are shown as mean values ± standard deviations (SD). **(D)** Cell viability after rRABVs infection. BSR cells were infected with different rRABVs at MOI of 0.01 or DMEM (mock infection). Cell activities were detected at 1, 2, 3, or 4 dpi with a commercial cell activity kit. All samples were undertaken in quadruplicate, and the results are shown as the mean values ± SD. **(E)** Production of HMGB1^wt^ or HMGB1^mut^ was detected by indirect immunofluorescence assay (IFA). BSR cells were infected with different rRABVs at MOI of 0.01 or DMEM (mock infection). Two days later, the culture mediums were removed and the cells were fixed, permeated, blocked, and then stained with HMGB1 antibody, fluorescent antibody, and DAPI. The results were detected by fluorescence microscope. Red arrows indicate the expression of HMGB1. **(F)** Production of HMGB1^wt^ or HMGB1^mut^ was detected by western blotting. BSR cells were infected with LBNSE-HMGB1^wt^, or LBNSE-HMGB1^mut^ at MOI of 0.01, 0.1, or with LBNSE at MOI of 1, or mock infected with DMEM. After incubation at 37°C for 24 h, the cells were collected and lysed for western blotting analysis, and β-actin was used as an internal standard. **(G)** Production of HMGB1^wt^ or HMGB1^mut^ in the culture medium was detected by western blotting. BSR cells were infected with different rRABVs at MOI of 1 or mock infected with DMEM. The culture supernatants were harvested for western blotting.

HMGB1 was demonstrated to promote DCs activation, to further investigate the role of DCs activation by HMGB1 in immunogenicity of RABV, HMGB1 was cloned into the genome of RABV LBNSE strain in this study. The wild-type HMGB1 (HMGB1^wt^) is mainly localized in the nucleus, and to achieve the purpose of secretion for enhancing the potent adjuvant activity, all the serine residues within both nuclear localization signals (NLSs) of HMGB1 were mutated to alanine residues and an immunoglobulin E (IgE) leader sequence was also inserted (designated as HMGB1^mut^) according to the previous study [30] (Figure 1A). The HMGB1^wt^ and HMGB1^mut^ gene were then individually cloned into the genome of RABV LBNSE strain between G and L gene, designated as LBNSE-HMGB1^wt^ and LBNSE-HMGB1^mut^, respectively (Figure 1B). The insertion of HMGB1^wt^ and HMGB1^mut^ gene was confirmed by sequencing and these recombinant rabies viruses (rRABVs) were rescued in BSR cells as described previously [14]. In order to characterize the two rRABVs *in vitro*, multi-step growth kinetic and cell activity assay were carried out in BSR cells. As shown in Figure 1C, the growth curve of LBNSE-HMGB1^wt^ and LBNSE-HMGB1^mut^ in BSR cells were similar with the parent strain LBNSE, although a slight decrease was observed (approximate 0.5 Log FFU/ml), indicating that the insertion of HMGB1^wt^ or HMGB1^mut^ did not significantly affect the viral replication *in vitro*. For the cell activity, no significantly change was observed in cells inoculated with the rRABVs compared with mock-infection cells at all tested time points (Figure 1D). To examine the expression of HMGB1^wt^ and HMGB1^mut^, indirect immunofluorescence assay (IFA) and western blotting assay were conducted. As expected, HMGB1^wt^ and HMGB1^mut^ were successfully expressed in the BSR cells infected with each rRABV, and HMGB1^wt^ was mainly observed in the nucleus, while HMGB1^mut^ was also detected in cytoplasm of LBNSE-HMGB1^mut^ infected BSR cells as shown in Figure 1E (pointed with red arrows). This result indicated that the mutations in both NLSs of HMGB1 and insertion of IgE leader sequence successfully affected the expression pattern of HMGB1 as designated, and it was further confirmed by the following western blotting assay. As shown in Figure 1F, HMGB1^wt^ and HMGB1^mut^ were expressed in a dose-dependent manner in cell lysate of LBNSE-HMGB1^wt^ and LBNSE-HMGB1^mut^ infected cells, respectively, and the expression level of HMGB1^mut^ was lower than HMGB1^wt^ in the same infective dose (ID) according to the ratio for expression level of HMGB1/β-actin. Moreover, as shown in Figure 1G, HMGB1^mut^ was detected in culture medium of LBNSE-HMGB1^mut^ infected cells, while no HMGB1^wt^ expression was observed in the LBNSE-HMGB1^wt^ or LBNSE infected or uninfected cell culture medium, suggesting that LBNSE-HMGB1^mut^ could enhance the secretion of HMGB1 in infected cells. Collectively, these data indicate that LBNSE-HMGB1^wt^ and LBNSE-HMGB1^mut^ were successfully constructed, and HMGB1^wt^ was well expressed and mainly localized in nucleus, while HMGB1^mut^ could be well secreted in infected cells as expected.

### *In vitro* activation of BMDCs after infection with rRABVs

**Figure 2:**
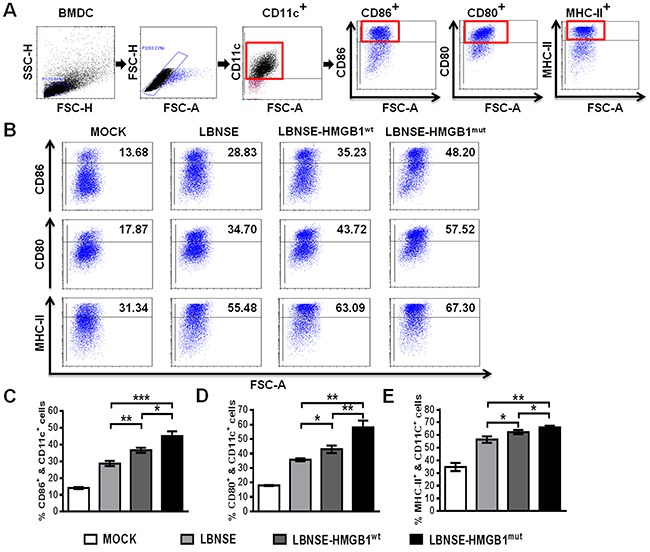
Activation of BMDCs *in vitro* after infection with different rRABVs Femur bone marrow was acquired from BALB/c mice, and BMDC precursors were induced by GMCSF and IL-4. The precursor cells were infected with each rRABV at MOI of 1. The culture medium from untreated cells (DMEM) was used as a negative control. One day later, the activation of BMDCs was analyzed with flow cytometery. Representative gating strategies for detection of BMDCs **(A)** and representative flow cytometric plots of BMDCs **(B)** are presented. The detailed analysis for activated BMDCs (CD11c^+^ & CD86^+^, CD11c^+^ & CD80^+^ or CD11c^+^ & MHC-II^+^) **(C to E)** is shown. All data are shown as the mean values ± SD (n=3). The data was analyzed by an unpaired two-tailed t-test. The following notations were utilized to indicate significant differences between different groups for all experiments: ^*^, p<0.05; ^**^, p<0.01; ^***^, p<0.001; ns, not significant.

Previous studies demonstrated that HMGB1 promotes the activation of DCs [24, 28]. To investigate whether expression of HMGB1 in rRABV contributes to the activation of DCs *in vitro*, bone marrow-derived DCs (BMDCs) were isolated and infected with each rRABV, and three markers for DCs activation (CD86, CD80, and MHC-II) were employed to detect the activation of BMDCs. Gating strategies and representative flow cytometric plots for detecting activated BMDCs are as shown in Figure 2A and 2B, respectively. As presented in Figure 2C (CD11c^+^ & CD86^+^), Figure 2D (CD11c^+^ & CD80^+^) and Figure 2E (CD11c^+^ & MHC-II^+^), both LBNSE-HMGB1^wt^ and LBNSE-HMGB1^mut^ could activate significantly more BMDCs than parent virus LBNSE, and LBNSE-HMGB1^mut^ promoted significantly more activation of BMDCs than LBNSE-HMGB1^wt^. These data illustrate that expression of HMGB1 (either HMGB1^wt^ or HMGB1^mut^) by rRABV could promote the *in vitro* BMDCs activation compared with parent virus LBNSE, and secreted HMGB1 (HMGB1^mut^) is a better strategy for DCs activation *in vitro*.

### Recruitment and/or activation of DCs after immunization with rRABVs in mice

**Figure 3:**
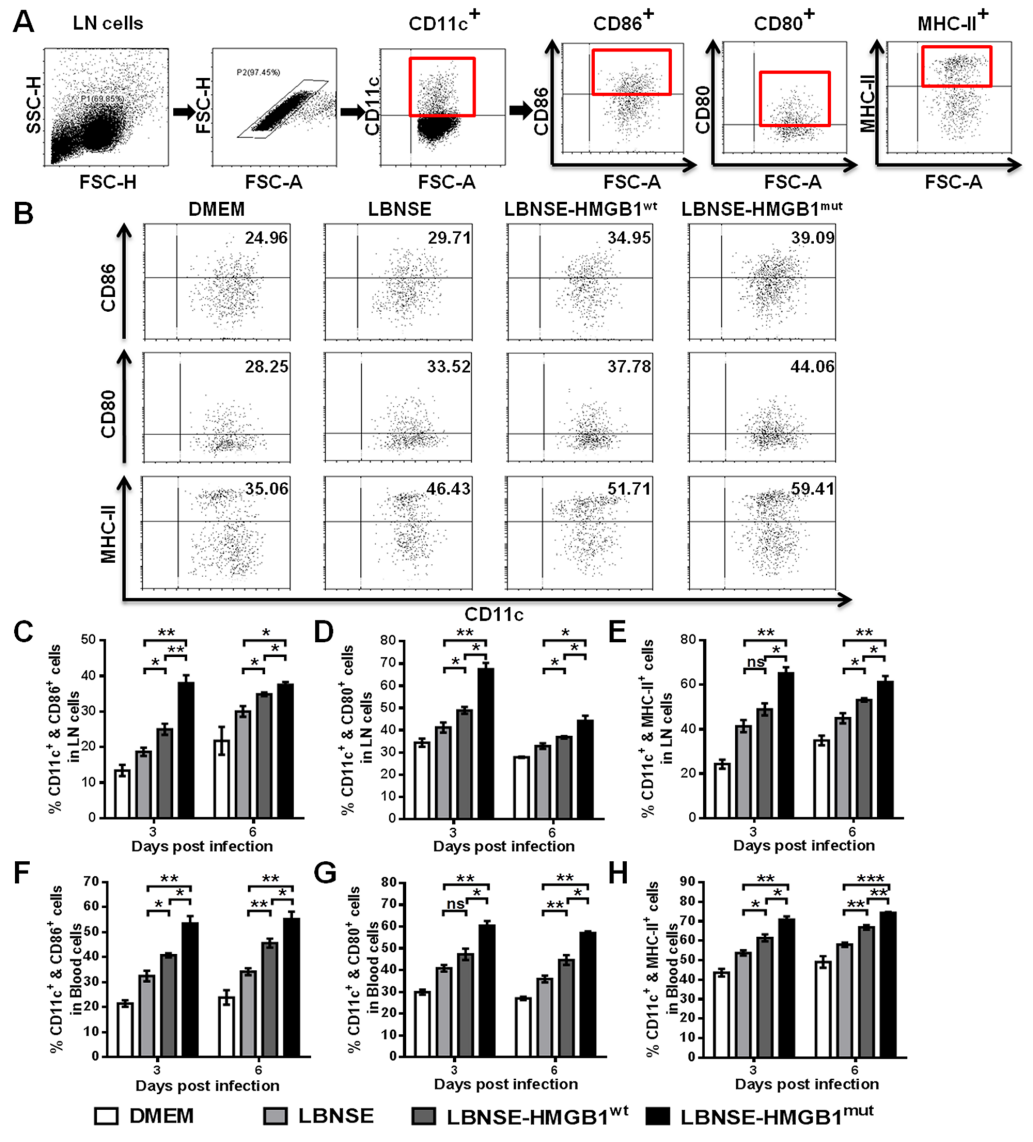
Recruitment and/or activation of DCs in mice immunized with different rRABVs BALB/c mice (n=3) were vaccinated by im injections of 1×10^6^ FFU of each rRABV or mock infected with DMEM. The draining (inguinal) lymph nodes (LNs) and blood were acquired at 3 and 6 dpi. Single-cell suspensions were prepared and incubated with antibodies against DCs and DCs activation markers, and then analyzed by flow cytometry. Representative gating strategies for the detection of DCs **(A)** and representative flow cytometric plots of DCs **(B)** from the draining LNs are presented. The detailed analysis for activated DCs (CD11c^+^ & CD86^+^, CD11c^+^ & CD80^+^ or CD11c^+^ & MHC-II^+^) from the draining LNs **(C, D, E)** and blood **(F, G, H)** at 3 and 6 dpi are shown. All data are shown as the mean values ± standard errors (SEM) (n=3). The data was analyzed by an unpaired two-tailed t-test. The following notations were utilized to indicate significant differences between different groups for all experiments: ^*^, p<0.05; ^**^, p<0.01; ^***^, p<0.001; ns, not significant.

Previous studies have indicated that HMGB1 can activate DCs and induce the migration DCs into draining Lymph Nodes (LNs) [29]. To investigate whether HMGB1^mut^ expressed by rRABV recruits and/or activates DCs *in vivo*, mice were immunized with 1×10^6^ FFU of each rRABV by intramuscular (im) route. Flow cytometry was performed to determine the activation of DCs (CD11c^+^ & CD86^+^, CD11c^+^ & CD80^+^, or CD11c^+^ & MHC-II^+^) in the draining (inguinal) LNs and peripheral blood at 3 and 6 days post immunization (dpi). The gating strategies and representative flow cytometric plots in inguinal LNs for detecting activated DCs are as presented in Figure 3A and 3B, respectively. Significantly more activated DCs were detected in the inguinal LNs (Figure 3C to 3E) and blood (Figure 3F to 3H) of mice immunized with LBNSE-HMGB1^wt^ than in those immunized with LBNSE at 6 dpi; notably, significantly more activated DCs were observed in the inguinal LNs (Figure 3C to 3E) and blood (Figure 3F to 3H) of mice vaccinated with LBNSE-HMGB1^mut^ than those vaccinated with LBNSE or LBNSE-HMGB1^wt^ at 3 and 6 dpi. Taken together, consistent with results of *in vitro* DCs activation by rRABVs infection, these data indicate that secretion of HMGB1^mut^ by LBNSE-HMGB1^mut^ could promote significantly more DCs activation in immunized mice than the mice immunized with parent virus LBNSE or LBNSE-HMGB1^wt^.

### Recruitment of Tfh cells after immunization with rRABVs in mice

**Figure 4:**
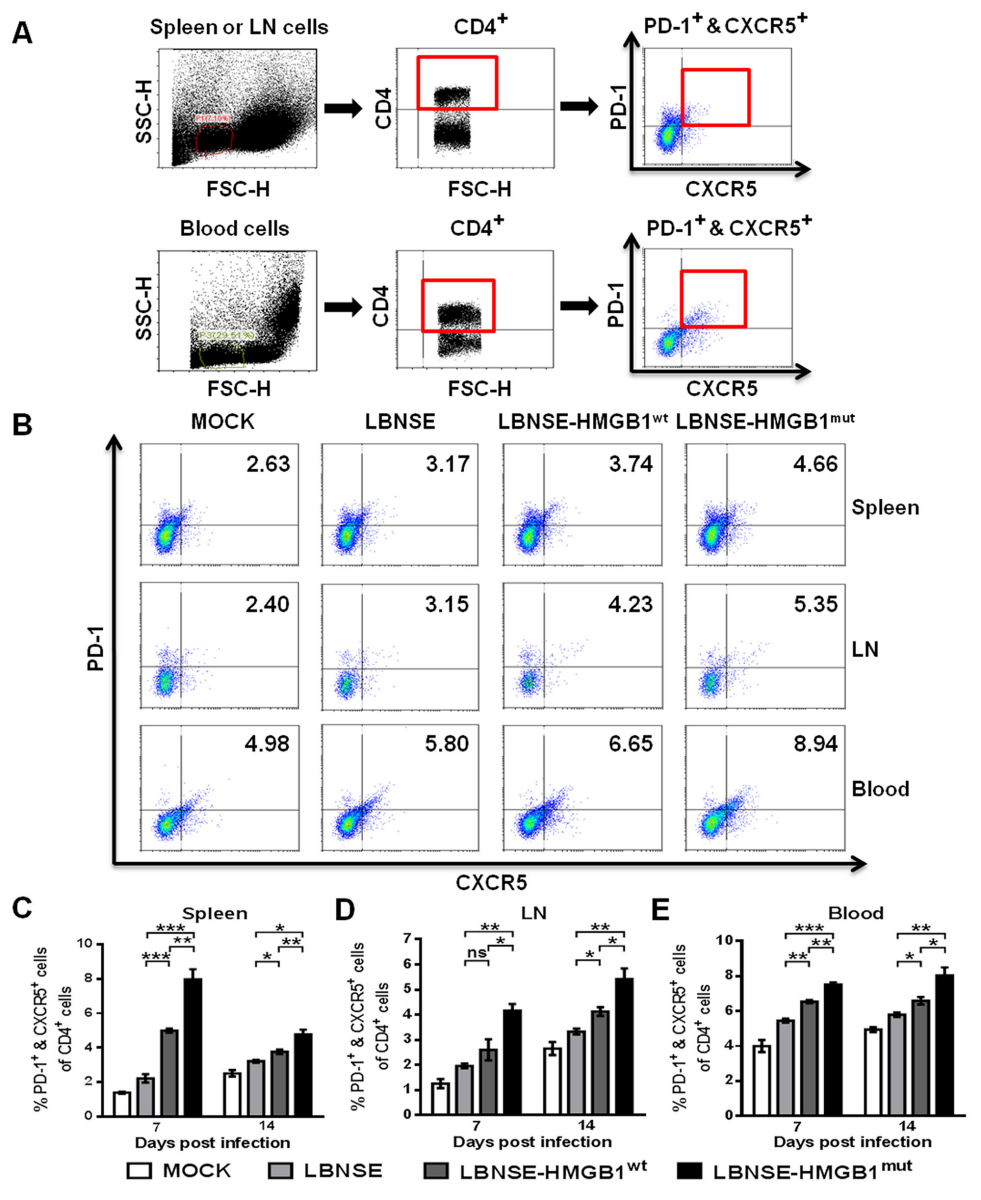
Recruitment of Tfh cells in mice immunized with different rRABVs BALB/c mice (n=3) were vaccinated with 1×10^6^ FFU of each rRABV or DMEM (mock infection) by im route. The spleens, draining LNs and blood were collected at 7 and 14 dpi. Single-cell suspensions were prepared and incubated with antibodies against Tfh cells and Tfh cell activation markers, and then analyzed by flow cytometry. Representative gating strategies for the detection of Tfh cells **(A)** and representative flow cytometric plots of Tfh cells at 14 dpi **(B)** are presented. Analysis for activated Tfh cells (PD-1^+^ & CXCR5^+^ of CD4^+^) at 7 and 14 dpi are shown for the spleen **(C)**, draining LNs **(D)** and blood **(E)**. All data are shown as the mean values ± SEM (n=3). The data were analyzed by an unpaired two-tailed t-test. The following notations were utilized to indicate significant differences between different groups for all experiments: ^*^, p<0.05; ^**^, p<0.01; ^***^, p<0.001; ns, not significant.

To investigate whether the expression of HMGB1^mut^ in rRABV increases the Tfh cells recruitment *in vivo*, mice were immunized via im route with 1×10^6^ FFU of each rRABV or mock immunized with an equal volume of DMEM, and flow cytometry was performed to quantify the Tfh cells (PD-1^+^ & CXCR5^+^ of CD4^+^) in the spleen, inguinal LNs and blood at 7 and 14 dpi. The gating strategies and representative flow cytometric plots for analyzing Tfh cells are as shown in Figure 4A and 4B, respectively. Significantly more Tfh cells were found in the spleens (Figure 4C), draining LNs (Figure 4D) and blood (Figure 4E) of mice vaccinated with LBNSE-HMGB1^mut^ than those vaccinated with LBNSE-HMGB1^wt^ or LBNSE at all tested time points (7 and 14 dpi); significantly more Tfh cells were detected in the spleens (Figure 4C), draining LNs (Figure 4D) and blood (Figure 4E) of mice vaccinated with LBNSE-HMGB1^wt^ than those vaccinated with LBNSE at all selected time points except for 7 dpi in inguinal LNs. Together, these results indicate that consistent with the results of DCs activation in immunized mice, mice vaccinated with LBNSE-HMGB1^mut^ could promote significantly more Tfh cells recruitment than those vaccinated with parent virus LBNSE or LBNSE-HMGB1^wt^.

### Recruitment of GC B cells after immunization with rRABVs in mice

**Figure 5:**
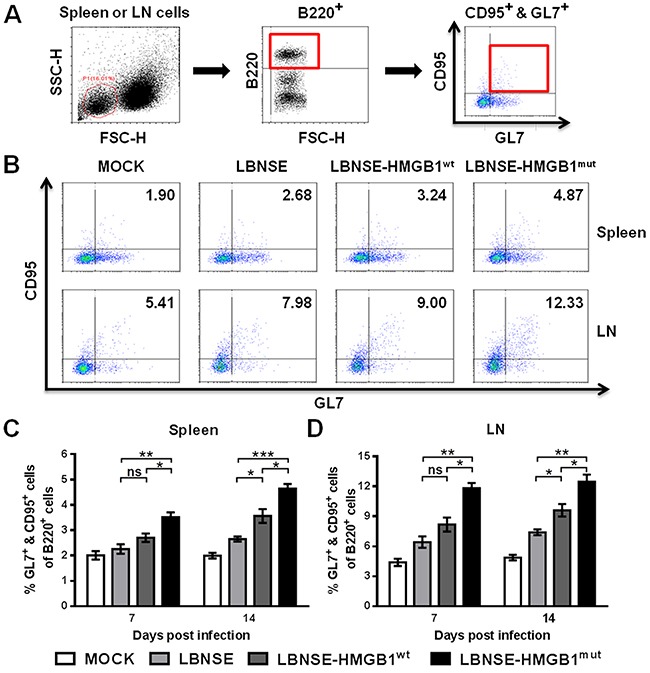
Recruitment of GC B cells in mice immunized with different rRABVs BALB/c mice (n=3) were vaccinated by im injections of 1×10^6^ FFU of each rRABV or DMEM (mock infection). The spleens and draining LNs were acquired at 7 and 14 dpi. Single-cell suspensions were prepared and incubated with antibodies against GC B cells and GC B cell activation markers, and then analyzed by flow cytometry. Representative gating strategies for the detection of GC B cells **(A)** and representative flow cytometric plots of GC B cells at 14 dpi **(B)** are presented. The detailed analysis for activated GC B cells (GL7^+^ & CD95^+^ of B220^+^) at 7 and 14 dpi are shown for the spleen **(C)** and draining LNs **(D)**. All data are shown as the mean values ± SEM (n=3). The data was analyzed by an unpaired two-tailed t-test. The following notations were utilized to indicate significant differences between different groups for all experiments: ^*^, p<0.05; ^**^, p<0.01; ^***^, p<0.001; ns, not significant.

It was found that the recruitment of Tfh cells could contribute to forming GC B cells [17, 21, 22]. Therefore, to investigate whether the expression of HMGB1 by rRABVs could increase the GC B cells, mice were vaccinated with 1×10^6^ FFU of each rRABV by im route, and the GC B cells (GL7^+^ & CD95^+^ of B220^+^) in the spleens and draining LNs were detected at 7 and 14 dpi by using flow cytometry. The gating strategies and representative flow cytometric plots for the detection of GC B cells are shown in Figure 5A and 5B, respectively. As expected, significantly more GC B cells were detected in the spleens (Figure 5C) and draining LNs (Figure 5D) of mice vaccinated with LBNSE-HMGB1^mut^ than those vaccinated with LBNSE or LBNSE-HMGB1^wt^ at 7 and 14 dpi; meanwhile, significantly more GC B cells were detected in the spleens (Figure 5C) and inguinal LNs (Figure 5D) of mice vaccinated with LBNSE-HMGB1^wt^ than those vaccinated with LBNSE at 14 dpi. Together, mice immunized with LBNSE-HMGB1^mut^ promoted significantly more GC B cells recruitment than those immunized with LBNSE or LBNSE-HMGB1^wt^.

### Generation of plasma cells after immunization with rRABVs in mice

**Figure 6:**
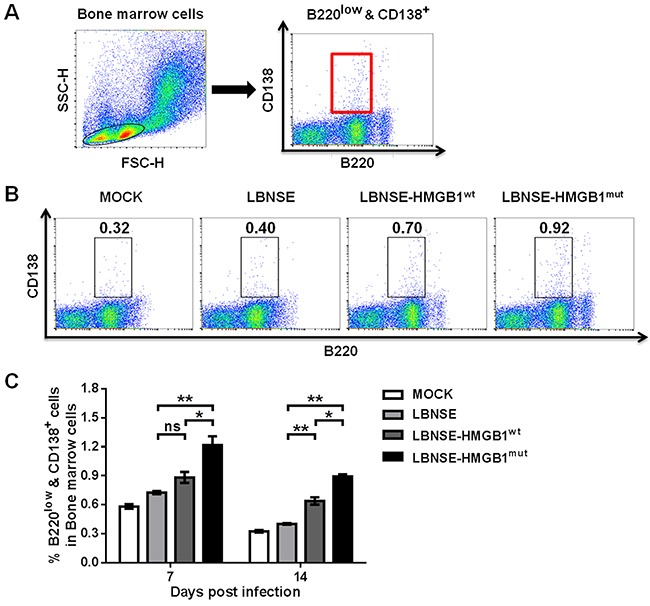
Generation of plasma cells in mice immunized with different rRABVs BALB/c mice (n=3) were vaccinated by im injections of 1×10^6^ FFU of each rRABV or DMEM (mock infection), and femur bone marrow samples were acquired at 7 and 14 dpi. Single-cell suspensions were prepared and incubated with antibodies against plasma B cells and plasma B cell activation markers, and then analyzed via flow cytometry. Representative gating strategies for the detection of plasma B cells **(A)** and representative flow cytometric plots of plasma B cells at 14 dpi **(B)** are presented. The detailed analysis of activated plasma B cells (B220^low^ & CD138^+^) at 7 and 14 dpi are shown for the bone marrow samples **(C)**. All data are shown as the mean values ± SEM (n=3). The data was analyzed by an unpaired two-tailed t-test. The following notations were utilized to indicate significant differences between different groups for all experiments: ^*^, p<0.05; ^**^, p<0.01; ^***^, p<0.001; ns, not significant.

GC B cells can further differentiate into plasma cells which lose the capacity to express chemokine C-X-C motif receptor 5 (CXCR5) [34]. Plasma cells then leave secondary lymphoid organs to reside primarily in bone marrow [34–38]. Hence, to investigate whether expression of HMGB1 by rRABVs could increase the generation of plasma cells *in vivo*, mice were vaccinated with 1×10^6^ FFU of each rRABV by im route. Femur bone marrow was acquired from immunized mice and flow cytometry was performed to determine the plasma cells (B220^low^ & CD138^+^). The gating strategies and representative flow cytometric plots for the detection of the plasma cells are as presented in Figure 6A and 6B, respectively. As shown in Figure 6C, Significantly more plasma cells were found in mice vaccinated with LBNSE-HMGB1^mut^ than those vaccinated with LBNSE (P<0.01) or LBNSE-HMGB1^wt^ (P<0.05) at 7 and 14 dpi, and significantly more plasma cells were observed in mice vaccinated with LBNSE-HMGB1^wt^ than in LBNSE vaccinated mice (P<0.01) at 14 dpi. These data demonstrate that secretion of HMGB1^mut^ by LBNSE-HMGB1^mut^ could promote significantly more plasma cells generation in immunized mice than those immunized with LBNSE or LBNSE-HMGB1^wt^.

### Pathogenicity and immunogenicity of the rRABVs in mice

**Figure 7:**
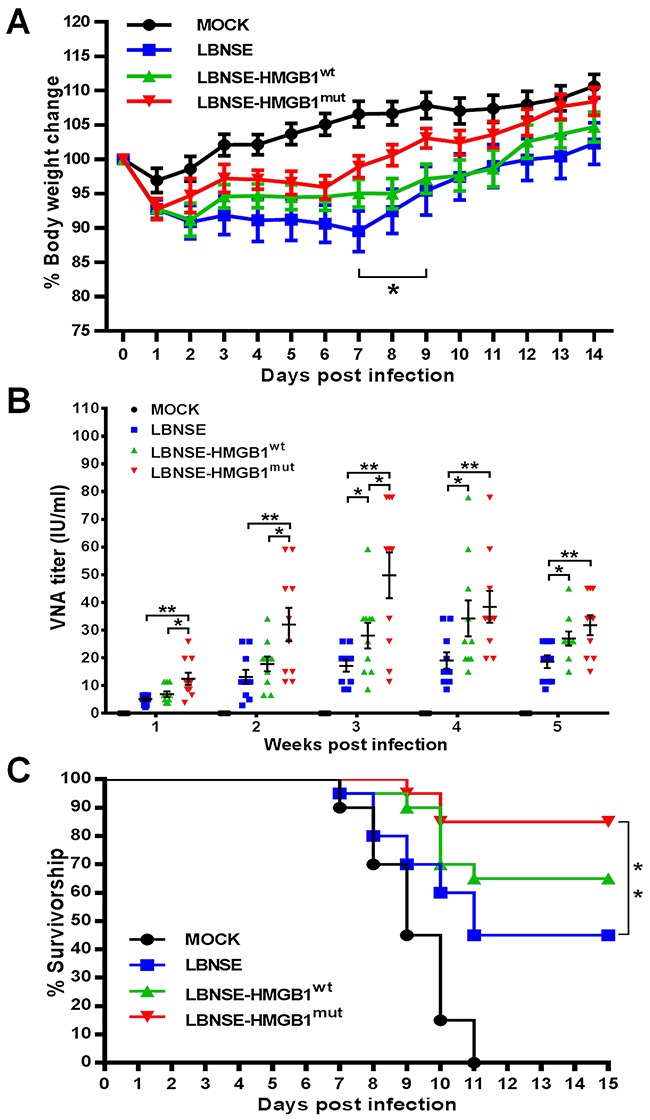
Pathogenicity and immunogenicity of different rRABVs in mice ICR mice were vaccinated with 1×10^6^ FFU of each rRABV or DMEM (mock infection) by ic route, and the body weights were measured and recorded daily for 2 weeks **(A)**. Data are shown as the mean values ± SEM (n=9 or 10). **(B)** Groups of ICR mice (n=10) were vaccinated with 1×10^6^ FFU of rRABVs or DMEM (mock infection) by im route. At indicated time points post immunization, blood samples were acquired and VNA titers were tested via FAVN tests. Titers were normalized to international units according to the WHO standard and are shown as mathematic mean titers. **(C)** Groups of ICR mice (n=20) were vaccinated with 1×10^6^ FFU of rRABVs by im route. Two weeks after vaccination, the mice were challenged with 50 LD_50_ of pathogenic RABV strain CVS-24 by ic route and monitored for another 2 weeks, and survivorship was taken notes. Kaplan-Meier survival curves were analyzed by the log rank test for the percent survival experiments. The other data analyzed by an unpaired two-tailed t-test. The following notations were utilized to indicate significant differences between different groups for all experiments: ^*^, p<0.05; ^**^, p<0.01; ^***^, p<0.001; ns, not significant.

To determine whether the expression of HMGB1 could affect the pathogenicity of RABV *in vivo*, 6 weeks old female ICR mice were infected intracerebral (ic) with a high dose (4×10^6^ FFU) of different rRABVs. The mice were monitored daily for 2 weeks to estimate disease development and weight loss; neither death nor clinical neurological symptoms were observed during the 2-week monitoring period. For body weight change, mice vaccinated with LBNSE-HMGB1^mut^ (92.75%) showed similar weight loss with mice infected with LBNSE-HMGB1^wt^ (92.81%) or LBNSE (92.76%) at 1 dpi; the trend of weight loss for LBNSE-HMGB1^wt^ infected mice was similar to that of LBNSE vaccinated mice within 2 weeks; of note, it was found that the weight loss in mice infected with LBNSE-HMGB1^mut^ (98.94%) were significantly lower than that of mice injected with LBNSE (89.54%) from 7 to 9 dpi (P<0.05) as shown in Figure 7A. These data indicate that the expression of HMGB1 by rRABV did not enhance the pathogenicity in mice, and expression of HMGB1^mut^ could transiently decrease the pathogenicity, which would be further investigated for the mechanism in future study.

To determine whether expression of HMGB1 by rRABVs could enhance the immunogenicity, mice were vaccinated with 1×10^6^ FFU of different rRABVs by im route. Blood samples were collected weekly, and sera were isolated for the determination of virus-neutralizing antibody (VNA) using the fluorescent antibody virus neutralization (FAVN) tests. As shown in Figure 7B, significantly higher VNA titers were detected in mice vaccinated with LBNSE-HMGB1^mut^ (12.45 IU/ml) than mice vaccinated with LBNSE at all chosen time points (P<0.01) or vaccinated with LBNSE-HMGB1^wt^ from 1 to 3 wpi (p<0.05); in addition, significantly higher levels of VNA were detected in mice vaccinated with LBNSE-HMGB1^wt^ than those vaccinated with LBNSE from 3 to 5 wpi (p<0.05). The highest levels of VNA detected in mice immunized with each rRABV were 49.81 IU/ml (at 3 wpi), 34.20 IU/ml (at 4 wpi), and 19.70 IU/ml (at 4 wpi) for LBNSE-HMGB1^mut^, LBNSE-HMGB1^wt^, and LBNSE, respectively. To further investigate whether the higher VNA titers were correlated to better protection, mice were challenged with 50 times of 50% lethal dose (LD_50_) of pathogenic RABV strain CVS-24 through ic route at 14 dpi. The mice were monitored for another 2 weeks and the survivorship in each group was recorded. As shown in Figure 7C, all the mock immunized mice died till 11 dpi, and significantly more mice immunized with LBNSE-HMGB1^mut^ were protected (85%) than those vaccinated with LBNSE (45%) (P<0.01) or LBNSE-HMGB1^wt^ (65%) (no significant difference). Taken together, all these data indicate that LBNSE-HMGB1^mut^ quickly promotes VNA production as early as 1 wpi and offers better protection than LBNSE or LBNSE-HMGB1^wt^, indicating LBNSE-HMGB1^mut^ could be a promising rabies vaccine strain.

## DISCUSSION

Dendritic cells (DCs), the major antigen-presenting cell (APC), link the innate recognition of viruses to the adaptive immune response, and play a vital role in the presentation of the vaccine antigens to the immune system [39]. Regulatory DCs are responsible for B cell differentiation [40] and a small numbers (250 to 1,000) of DCs can directly stimulate the proliferation of activated B cells, and then promote the production of antibody [34]. Our previous studies have found that recombinant attenuated RABV expressing chemokines or cytokines could enhance the immunogenicity by recruiting and/or activating DCs, and then promote the adaptive immune response in organism [12–15, 41]. Following this rationale, the rRABV expressing an optimized HMGB1 (LBNSE-HMGB1^mut^) constructed in this study also could enhanced the adaptive immune response in immunized mice by activating DCs and further recruiting the Tfh, GC B and plasma cells.

Host-derived molecules, termed damage-associated molecular patterns (DAMPs, also called alarmins), activate the pattern recognition receptors (PRRs), which in turn stimulate cytokine release and activate the innate immune system [42]. DAMPs can promote phagocytosis, antigen-presentation, and inflammasome activation in DCs, collectively fostering T cell priming against antigens [43]. HMGB1 is a prototypical DAMP that mediates immune responses in the noninfectious inflammatory response [23] and promotes the maturation and activation of DCs [24, 28, 44]. HMGB1 can induce the migration of DCs into draining Lymph nodes (LNs) by interacting with its receptor advanced glycation endproducts (RAGE) [29] and is an endogenous immune adjuvant [33]. Notably, recent studies have indicated that HMGB1 is an effective immune adjuvant molecule which enhances the humoral immune response of influenza [31] and HIV [30] in DNA vaccination. It has also been demonstrated that HMGB1 acts as an adjuvant for tuberculosis subunit vaccines [32]. Remarkably, these studies were only found that HMGB1 could enhance the immune response by activating DCs, and our previous studies usually detected the activated DCs and the total B cells [11–15]; however, in the present study, the following process of humoral immune response for RABV vaccination after DCs activation were further investigated; we found that rRABV expressing optimized HMGB1 (HMGB1^mut^ or HMGB1^wt^) could enhance the humoral immune response by activating DCs, and then recruiting Tfh cells, GC B cells and generating plasma cells. Moreover, it was the first time approved that HMGB1 was a promising adjuvant in an attenuated virus vaccine without significantly affecting the viral replication.

Nuclear localization signals (NLSs) in proteins determine their binding to nuclear importing proteins and consequently influence their nuclear accumulation. HMGB1 has two NLSs and two putative nuclear export signals (NESs) to control the nuclear transport [45]. The acetylation of both NLSs of HMGB1 arerelated to nuclear export toward secretion [46]. Previous study has demonstrated that the mutation of all serine residues to alanine residues in both NLSs could decrease the binding to nuclear importing protein and permits relocation to the cytoplasm and subsequent secretion [30]. Based on this, an HMGB1^mut^ with mutations of all the serine to alanine in both NLSs and insertion of an IgE signal sequence was constructed. Indeed, HMGB1^mut^ produced by LBNSE-HMGB1^mut^ could secret outside the cells since it was detected in cell culture medium as shown in Figure 1G. Interestingly, over-expression of HMGB1^mut^ slightly restricted the replication of RABV (0.5 Log FFU/ml) and also transiently reduced body weight loss in immunized mice from 7 to 9 dpi as shown in Figure 1C and 7B, respectively; however, the mechanism for the phenomenon is not clear. We tried to find whether HMGB1 could interact with viral proteins (N, P, M, or G) by immunoprecipitation assay, but no interaction between HMGB1 and RABV proteins was observed, indicating that HMGB1 may not directly interact with viral proteins to affect viral replication (data are not shown). Further studies are warranted to figure out the mechanism for HMGB1 restricting RABV replication and reducing the pathogenicity after immunization. Release of HMGB1 was observed during many pathogens infections, such as hepatitis B virus (HBV) [47], HIV-1 [48], and mycobacterial [49]. Recent study indicated that porcine epidemic diarrhea virus (PEDV) infection also contributed to HMGB1 transcription and release [50]. However, in our study, no over-expression and/or secretion of HMGB1 were detected in parent virus LBNSE infected cells, suggesting RABV infection did not enhance HMGB1 releasing (at least in BSR cells).

Tfh cells, a subset of CD4^+^ T cells, specially providing help to B cells, are essential for GC reaction, affinity maturation, and the development of most high affinity antibodies and memory B cells [17]. The most important function of Tfh cells is to help GC development and the major site of B cell affinity maturation in the GC [17]. Tfh cells regulate the size of GC, limit low affinity B cells entry into the GC, sustain high affinity B cells occupancy of the GC, and select high affinity B cells during affinity maturation [17]. Regulation of Tfh cell help is central for achieving the goal of GC responses, which is to generate and select GC B cells with higher affinity for the pathogens [51]. Previous study has found that the rRABV expressing GM-CSF could activate DCs, and then increase the recruitment of Tfh, GC B and plasma cells [41]. Similarly, in this study, over-expression of HMGB1^mut^ enhance the recruitment of Tfh, GC B cells in mice. As is known, GC B cells can differentiate into long-lived memory plasma cells and long-lived B cells. As expected, more plasma cells were found in the bone marrow of mice immunized with LBNSE-HMGB1^mut^ than those immunized with LBNSE or LBNSE-HMGB1^wt^. The strategy that using rRABVs expressing cytokines or chemokines to promote the activation of immune cells, including DCs and B cells is demonstrated to result in greater production of VNA and better protection [13, 15, 52]. Consistent with these studies, mice vaccinated with LBNSE-HMGB1^mut^ quickly induced the production of VNA and offered a better protection against lethal RABV challenge than LBNSE or LBNSE-HMGB1^wt^.

In summary, this study indicates that the rRABV expressing HMGB1^mut^ could promote significantly more DCs activation, and recruitment of Tfh, GC B, and plasma cells than the parent virus LBNSE or LBNSE-HMGB1^wt^, and consequently provides a better protection by elevating the production level of VNA. These findings offer us a better understanding for the role of HMGB1 in RABV-induced humoral immune responses, and suggest that LBNSE-HMGB1^mut^ has the potential to be developed as a promising rabies vaccine.

## MATERIALS AND METHODS

### Cells, viruses, recombinant proteins, antibodies and animals

BSR cells, a cloned cell line derived from BHK-21 cells, were maintained in Dulbecco's modified Eagle's medium (DMEM, Gibco) supplemented with 10% fetal bovine serum (FBS, Gibco), and mouse neuroblastoma (NA) cells were cultured in RPMI 1640 (Mediatech) containing 10% FBS. LBNSE is a recombinant RABV (rRABV) strain which is originated from attenuated RABV strain SAD-B19 [53, 54] via mutation of the G protein at amino acid (aa) positions 194 and 333 [13]. The challenge virus standard 11 (CVS-11) and 24 (CVS-24), two pathogenic RABV strains, were individually propagated in NA cells and suckling mouse brains, respectively. Recombinant mouse GM-CSF and IL-4 were purchased from Novoprotein Scientific, Inc. (Shanghai, China). Rabbit anti-mouse HMGB1 and rabbit anti-mouse β-actin antibodies were purchased from Abcam (Cambridge, England). Horseradish peroxidase (HRP)-conjugated goat anti-rabbit antibody was purchased from BOSTER (Wuhan, China). Fluorescein isothiocyanate (FITC)-conjugated anti-RABV N protein antibody was purchased from FujiRab (Melvin, PA). Antibodies used in flow cytometric analysis, including FITC anti-mouse CD11c (clone N418), FITC anti-mouse CD4 (clone GK1.5), FITC anti-mouse/human CD45R/B220 (clone RA3-6B2), PE anti-mouse CD86 (clone GL-1), PE anti-mouse CD279 (PD-1) (clone RMP1-30), PE/Cy7 anti-mouse I-A/I-E (MHC-II) (clone M5/114.15.2), APC anti-mouse CD80 (clone 16-10A1), APC anti-mouse CD185 (CXCR5) (clone L138D7), APC anti-mouse CD138 (Syndecan-1) (clone 281-2), and Alexa Fluor 647 anti-mouse/human GL7 (clone GL7), were all purchased from BioLegend (San Diego, CA), while PE Anti-Mouse CD95 (APO-1/Fas) (clone 15A7) were purchased from eBioscience (San Diego, CA). Female ICR and BALB/c mice (6 weeks old) were purchased from the Center for Disease Control (CDC) and Prevention of Hubei Province, China. All animal experiments were carried out as approved by the Scientific Ethics Committee of Huazhong Agricultural University (permit number HZAUMO-2015-016).

### Construction of rRABV clones

Mouse HMGB1 (HMGB1^wt^) cDNA was amplified from total RNA extracted from RABV-infected mouse brain using the SuperScript III One-Step reverse transcription (RT)-PCR system with Platinum Taq DNA polymerase (Invitrogen Life Technology). Two primers were used to acquire the HMGB1 gene (forward primer: 5′-TTGCGTACGGCCACCATGGGCAAAGGAGATCC-3′ and reverse primer: 5′-CTAGCTAGCTTATTCATCATCATCATC-3′, *Bsi*WI and *Nhe*I sites were underlined). The mutant type of HMGB1 (HMGB1^mut^) was synthesized from TSINGKE (Wuhan, China). The PCR product and Synthetic gene were digested with *Bsi*WI and *Nhe*I (New England Biolabs, Berverly, MA). After recycling, the genes were ligated into the rRABV vector LBNSE between G and L gene, which was described previously [13, 41].

### Rescue of rRABVs

Recombinant RABVs were rescued as described previously [55] and propagated in BSR cells. In brief, BSR cells were transfected with 2.0 μg of full infectious clone, 0.5 μg of pH-N, 0.25 μg of pH-P, 0.15 μg of pH-G, 0.1 μg of pH-L using the SuperFect transfection reagent (Qiagen, Valencia, CA) in accordance with the manufacture's protocol. At four days post transfection, the culture supernatant was replaced with fresh medium and incubated for another three days, and then the culture medium was collected and subjected to detect the rescued rRABV using FITC-conjugated anti-RABV N antibodies (Abs).

### Virus titration

rRABVs were titrated in BSR cells using direct fluorescent antibody assays as described previously [41]. In brief, BSR cells (in 96-well plates) were inoculated with serial 10-fold dilutions of each rRABV, and then incubated at 37°C for 3 days. The culture medium was discarded, and then the cells were washed 3 times with phosphate-buffered saline (PBS) and fixed with 80% ice-cold acetone at -20°C for 30 min. The cells were stained with FITC-conjugated anti-RABV N protein Antibodies for 1 h at home temperature. After 3 washes in PBS, antigen-positive foci on the cells were counted via an Olympus IX51 fluorescence microscope, and viral titers were calculated as fluorescent focus units per milliliter (FFU / ml). All titrations were carried out in quadruplicate.

### Cell viability assay

BSR cells seeded in 96-well plates were non-infected or infected with different rRABVs at MOI of 0.01. The cell viability was detected at different time points via CellTiter 96 Aqueous One Solution Cell Proliferation Assay (Promega) according to the protocol. In brief, the reagent were added into the chambers of 96-well plates, and incubated at 37°C for 1-4 h in an incubator with 5% CO_2_. Then the absorbance value of 490 nm was detected with the Microplate Reader (Molecular Devices, China).

### Indirect immunofluorescence assay (IFA)

BSR cells seeded in 24-well plates were non-infected or infected with different rRABVs at MOI of 0.01. Two days later, the cells were fixed for 1 h with 2% paraformaldehyde and permeated for 1 h with 1% Triton X-100. Then, the cells were blocked by 2% fetal bovine serum (FBS). The cells were incubated with rabbit anti-mouse HMGB1 antibody, and subsequently stained with Alexa Fluor 488-conjugated goat anti-rabbit antibody. 4', 6-diamidino-2-phenylindole (DAPI) (1 μg/ml) was used to dye the nucleus. The cells were washed three times with PBS after incubation with each above mentioned antibody. Fluorescent images were captured under an Olympus 1×51 fluorescence microscope.

### Western blotting

BSR cells were either non-infected or infected with different rRABVs and then lysed with RIPA buffer (Thermo Scientific). The lysates incubated on ice for 20 min and mixed every 5 min. Then, the lysates were centrifuged at 12,000 g for 20 min to remove debris and the concentrations of proteins were quantified using a Protein Quantitative Kit (Beyotime, China). The proteins were diluted to equal concentrations and the same quantities of different samples were mixed with Laemmli Buffer (BIO-RAD). Proteins were resolved by 10% SDS-PAGE, transferred to a polyvinylidene fluoride (PVDF) membrane and blocked using non-fat milk powder overnight at 4°C. Then, the PVDF membrane was incubated with rabbit anti-mouse HMGB1 antibody or anti-mouse β-actin antibody, and incubated with goat anti-rabbit antibody labeled with horseradish peroxidase (HRP). When each above step was completed, the cells were washed three times with PBS. Finally, the PVDF membrane was detected via a Protein Imaging System (SYNGENE, England).

### Isolation and cultivation of bone marrow-derived DCs

Bone marrow-derived dendritic cells (BMDCs) were isolated and cultured as described previously [13, 14, 56]. Briefly, BALB/c mice were euthanized and femur bone marrow was acquired by cutting and collecting the bone between femur and hip joints. Then, the bone marrow was transferred to a 6-well plate through flushing by a 10-ml syringe loaded with RPMI 1640 and dissociated into a single cell suspension. The BMDCs precursors were counted on a hemocytometer and adjusted to a density of 2×10^5^ cells per ml, then cultured in DCs medium (RPMI medium containing 0.1% 2-mercaptoethanol, 1×nonessential amino acids, and 1×sodium pyruvate) supplemented with 20 ng/ml recombinant mouse GM-CSF and 10 ng/ml recombinant mouse IL-4.

### Flow cytometry

Flow cytometry was used to detect the immune cells in the spleen, draining LNs (inguinal or cervical), peripheral blood, bone marrow, as well as BMDCs. In Brief, the mice were anesthetized, and the spleen, LNs and bone marrow were collected and homogenized to cell suspensions through a 40 μm nylon filter, and then washed 2 times with PBS. For the blood samples, blood was sampled, and the red blood cells were lysed with ACK lysis buffer (BioSource International, Inc., Camarillo, CA) for 1 min at room temperature. Single-cell suspensions were prepared and stained in 0.2% bovine serum albumin (BSA) with fluorescence-conjugated antibodies for 30 min at 4°C in the dark. After incubation, cells were washed twice in PBS containing 0.2% BSA and fixed in 1% paraformaldehyde in PBS for 30 minutes. Then the samples were performed via a BD LSR-II flow cytometer, and data was FlowJo software (TreeStar, San Carlos, CA) and BD FACS-Diva software (BD Pharmingen).

### Virus-neutralizing antibody (VNA) tests

Blood samples were collected and serum was isolated for the measurement of VNA by using the FAVN tests as described previously [41]. In brief, 50 μl of serial 3-fold dilutions of test and standard serum samples were added to 96-well microplates in 100-μl volumes. Each sample was added to four duplicated chambers. A 50-μl volume of CVS-11 suspension containing 50-200 FFU was added to each chamber. The microplates were then incubated at 37°C for 1 h with 5% CO_2_. Then, 50 μl of the BSR cells (5×10^5^ cells/ml) were added into each chamber, and the microplates were incubated at 34°C with 5% CO_2_ for 3 days. The plates were fixed in 80% ice cold acetone at -20°C for 30 min and then air-dried. Cells were stained with FITC-conjugated anti-RABV N antibodies for 45 min at 37°C, and then washed three times with PBS. The results were observed using an Olympus 1×51 fluorescence microscope. VNA titers were expressed in IU/ml based on comparisons with the titer of a reference serum obtained from the National Institute for Biological Standards and Control (Herts, UK) in each test.

### Ethics statement

All animal experiments were carried out in strict accordance with the protocols approved by The Scientific Ethic Committee of Huazhong Agricultural University (permit number: HZAUMO-2015-020). The animal care and maintenance were in compliance with the recommendations in the Regulations for the Administration of Affairs Concerning Experimental Animals of P.R. China.

### Statistical analysis

All data were analyzed by GraphPad Prism 6.0 software (La Jolla, CA). Kaplan-Meier survival curves were analyzed by the log rank test for the percent survival experiments. The other data statistical analyses were determined by an unpaired two-tailed t-test. The following notations were utilized to indicate significant differences between different groups for all experiments: ^*^, p<0.05; ^**^, p<0.01; ^***^, p<0.001.
